# Supplementary material for: Cooperative clamp-mediated promoter recognition by poxviral RNA polymerase and its TBP/TFIIB-like partner
Source: Nat Commun. 2026 Feb 18;17:1648. doi: 10.1038/s41467-026-69571-1 (PMC12917281; doi:10.1038/s41467-026-69571-1)
Supplement: Supplementary file 1 — Supplementory Information [file 41467_2026_69571_MOESM1_ESM.pdf]

Cooperative clamp-mediated promoter recognition by poxviral RNA polymerase and its TBP/TFIIB-like partner

Stefan Jungwirth[1†], Julia Bartuli[1], Stephanie Lamer[2], Andreas Schlosser[2], Clemens Grimm[1†*] and Utz Fischer[1,3‡*]

[1] Department of Biochemistry 1, Theodor Boveri-Institute, University of Würzburg, Am Hubland, 97074 Würzburg, Germany

[2] Rudolf Virchow Center for Experimental Biomedicine, University of Würzburg, 97080 Würzburg, Germany

[3] Helmholtz Institute for RNA-based Infection Research (HIRI), Helmholtz Center for Infection Research (HZI), 97080 Würzburg, Germany

[†] These authors contributed equally: Stefan Jungwirth, Clemens Grimm

[‡] Lead contact

* Correspondence: utz.fischer@uni-wuerzburg.de, clemens.grimm@uni-wuerzburg.de

Supplementary Figures

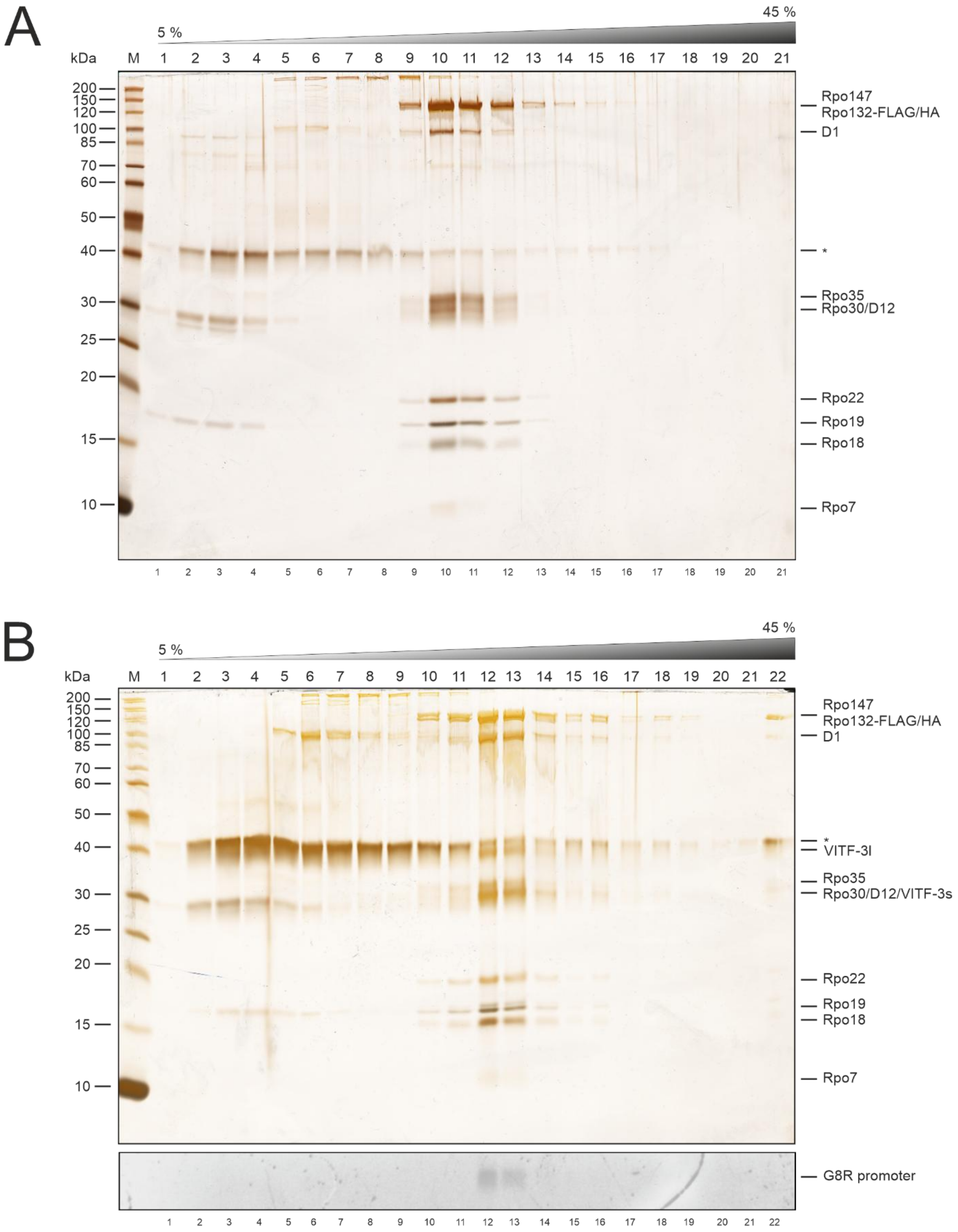

Supplementary Figure 1: Purification of vRNAP^{AraC} (A) and the iPIC (B) by sucrose density gradient centrifugation. A The FLAG-IP elution of HeLa S3 cells treated with AraC during GLV-1h439 infection was separated on a 5% to 45% sucrose density gradient. Fractions were analyzed by 12% SDS-PAGE and visualized by silver staining. The molecular weight marker is shown on the left and respective proteins are given on the right. The asterisk (*) indicates non-specifically co-purified actin. Source

data are provided as a Source Data file. **B** HeLa S3 lysate of AraC-treated cells during Vaccinia GLV-1h439 infection was incubated with intermediate promoter holding a DNA mismatch bubble from position −5 to +8. The sample was separated via 5% to 45% sucrose density gradient centrifugation, before fractions were analyzed on a 12% SDS-PAGE and visualized by silver staining. The lower panel shows ethidium bromide-staining in the same gel. The molecular weight marker is shown on the left and respective proteins are given on the right. The asterisk (*) indicates non-specifically co-purified actin. Source data are provided as a Source Data file.

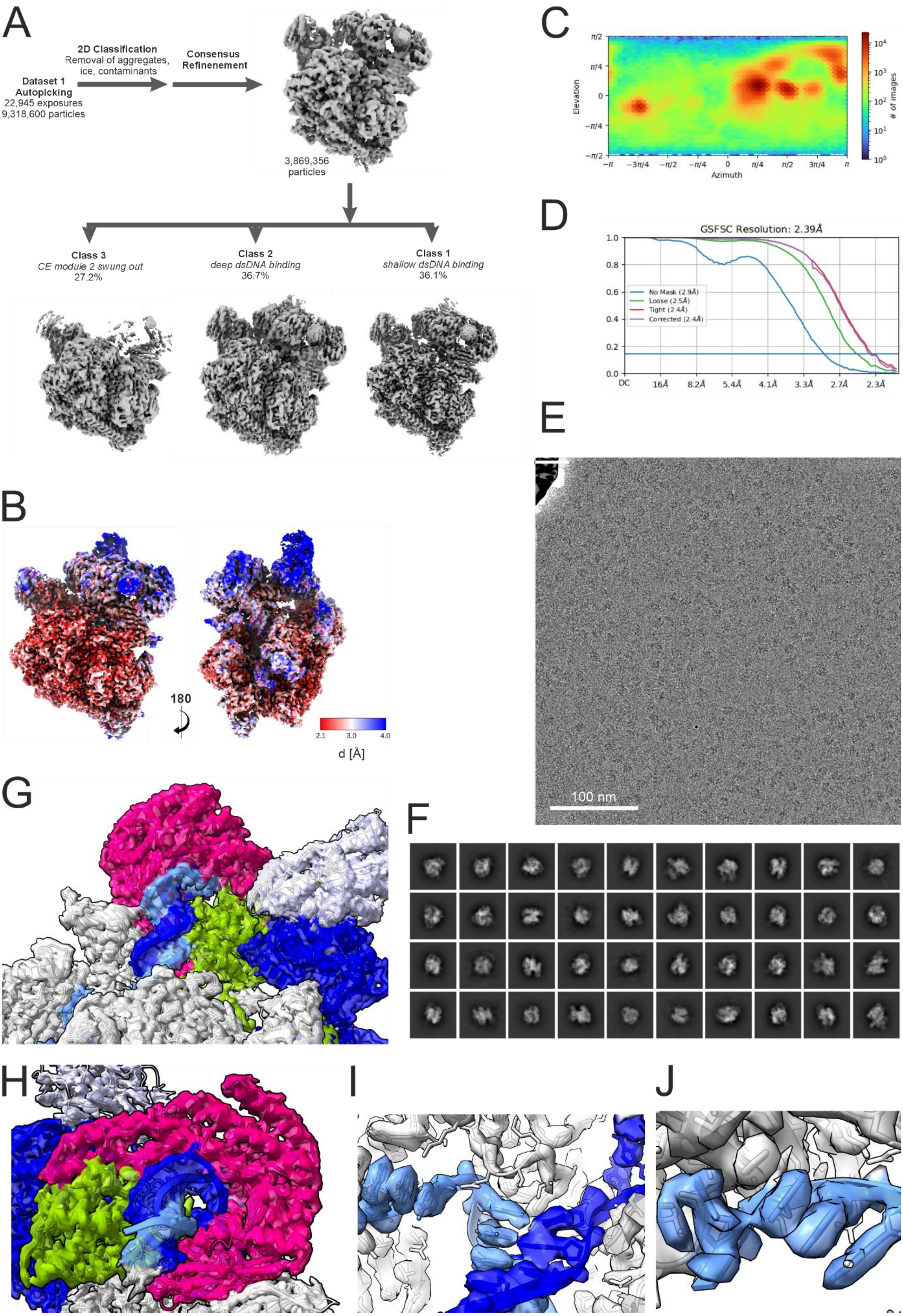

Supplementary Figure 2: Cryo-EM reconstruction of the iPIC. A Classification and refinement scheme of iPIC$_s$ (Class 1), iPIC$_d$ (Class 2) and iPIC$_{Cem}$ (Class 3). **B** Local resolution mapped to the reconstruction density isosurface for the iPIC$_d$ structure. The

local resolution of the cryo-EM map is color-coded and projected onto the density isosurface. **C** Orientation distribution plot for the final reconstruction of the $iPIC_d$ structure. **D** FSC plots of the final reconstruction of the $iPIC_d$. **E** Typical micrograph of the dataset with size marker indicating 100 nm. **F** Selected 2D classes. Panels **G–J** show surface views of the cryo-EM density (semi-transparent) with the fitted atomic model. **G** The VITF-3 region enclosing the promoter, viewed from downstream to upstream. **H** The same VITF-3 region viewed from upstream to downstream. **I** Close-up view of the downstream fork point. **J** Close-up view of the non-template strand at the initiator element.

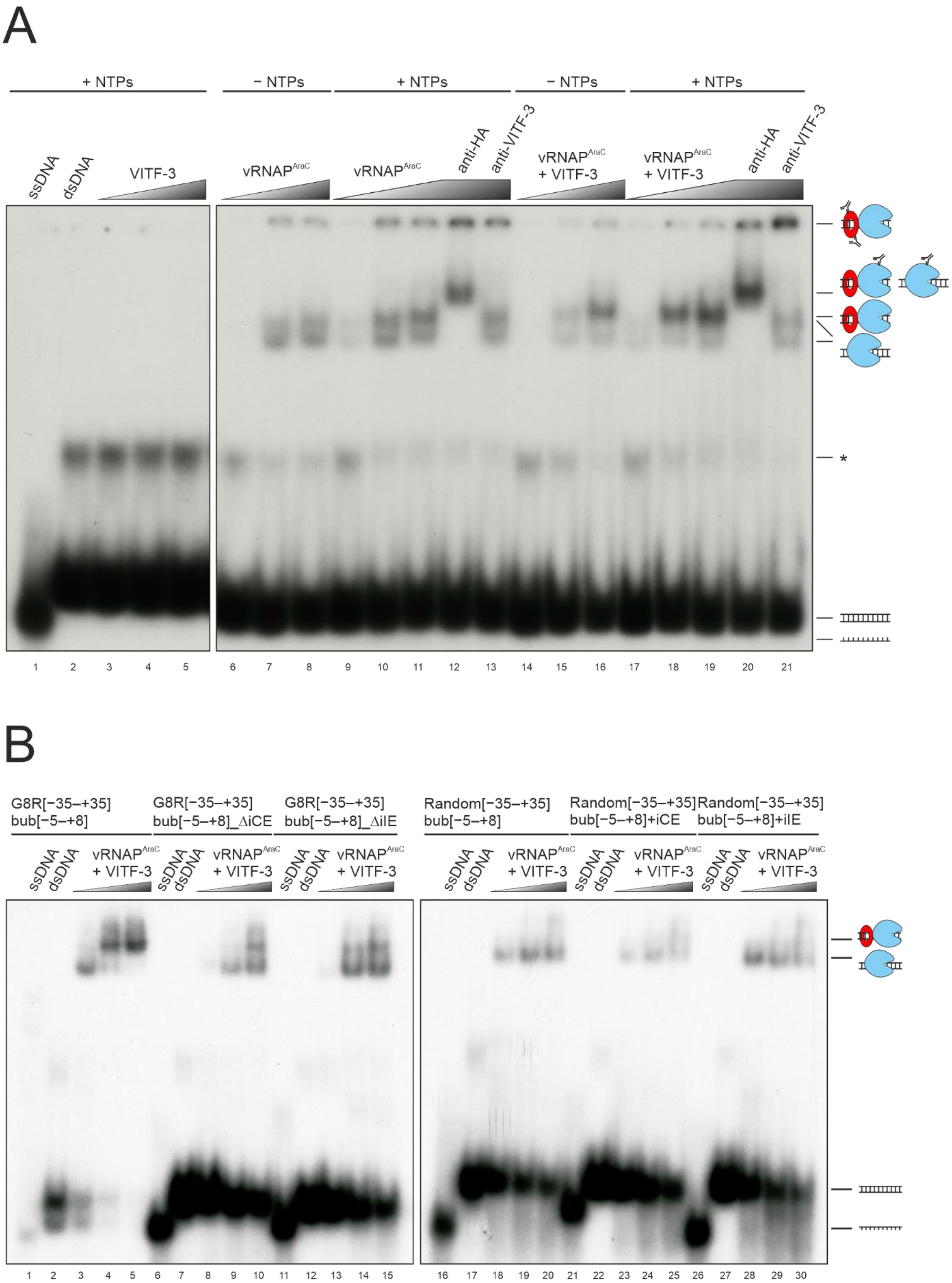

Supplementary Figure 3: EMSA reconstitution of the iPIC using different mismatch bubble scaffolds (A) or a native DNA template (B). EMSA results visualized by radio-autography. **A** The binding experiment was performed using a closed G8R promoter scaffold with a length of 70 bp. Addition of VITF-3, vRNAP^{AraC}, NTPs (1 mM each) and antibodies targeting HA (monoclonal; recognizing vRNAP^{AraC}) or VITF-3 (polyclonal), are indicated. DNA samples before (ssDNA) and after (dsDNA)

annealing are shown (lanes 1 and 2). Schematic representations of the resulting complexes are shown on the right. The asterisk (*) indicates an artifact of DNA annealing. Source data are provided as a Source Data file. **B** The experiments were conducted using radiolabeled 70 bp scaffolds containing DNA mismatch bubbles ranging from position −5 to +8. The scaffolds were based on the G8R promoter and two variants with either randomized core element (ΔiCE) or initiator element (ΔiIE), as well as on a randomized promoter and two variants with introduced core element (+iCE) or initiator element (+iIE). DNA samples before (ssDNA) and after (dsDNA) annealing are shown (lanes 1 and 2). Rectangles indicate increasing amount of recombinant VITF-3 and $vRNAP^{AraC}$. Utilized scaffolds and proteins are indicated on top and schematic representations of the generated complexes are shown on the right. Source data are provided as a Source Data file.

A

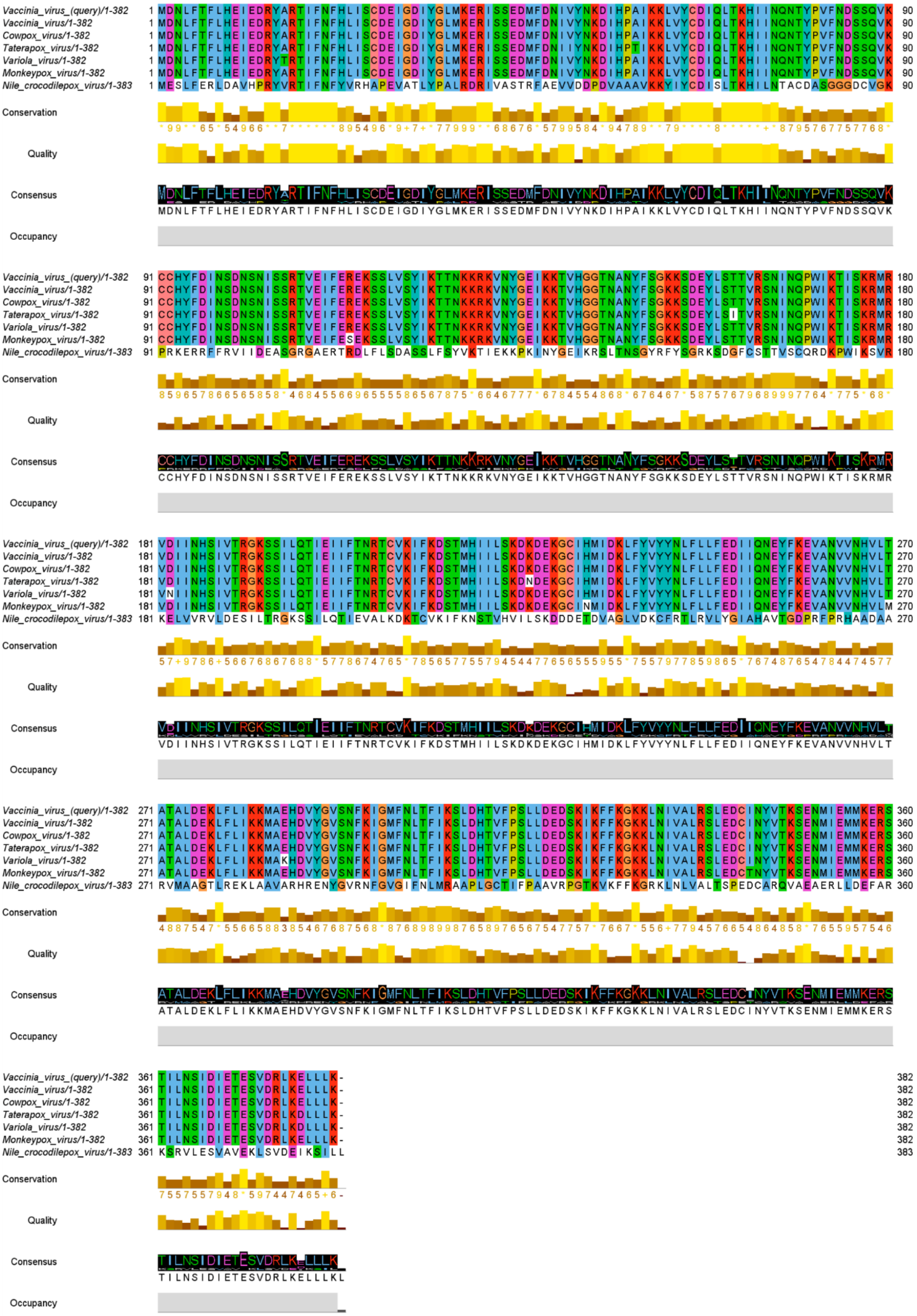

Vaccinia_virus_(query)/1-382
Vaccinia_virus/1-382
Cowpox_virus/1-382
Taterapox_virus/1-382
Variola_virus/1-382
Monkeypox_virus/1-382
Nile_crocodilepox_virus/1-383
Conservation
Quality
Consensus
Occupancy

B

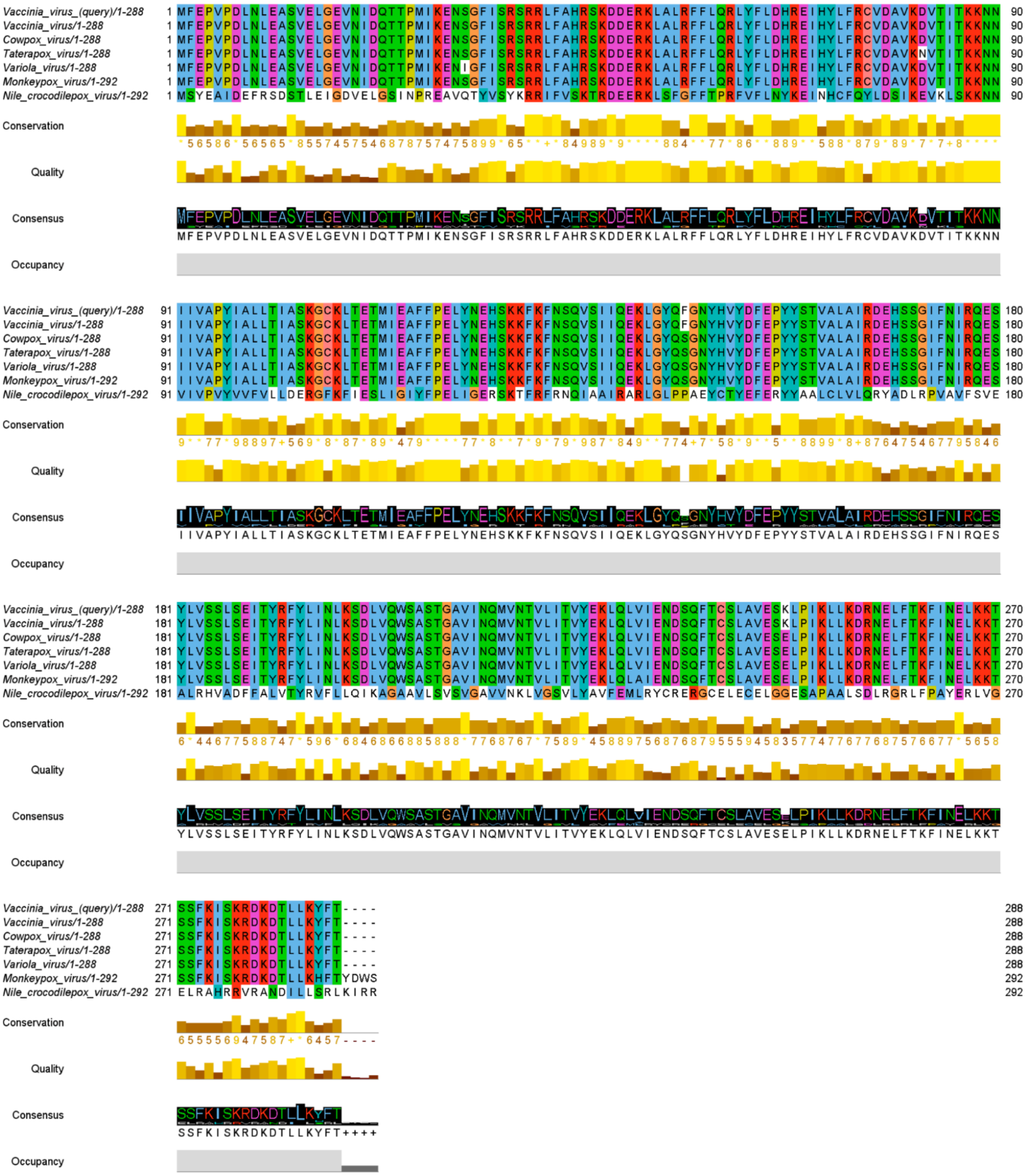
Vaccinia_virus_(query)/1-288
Vaccinia_virus/1-288
Cowpox_virus/1-288
Taterapox_virus/1-288
Variola_virus/1-288
Monkeypox_virus/1-292
Nile_crocodilepox_virus/1-292
Conservation
Quality
Consensus
Occupancy

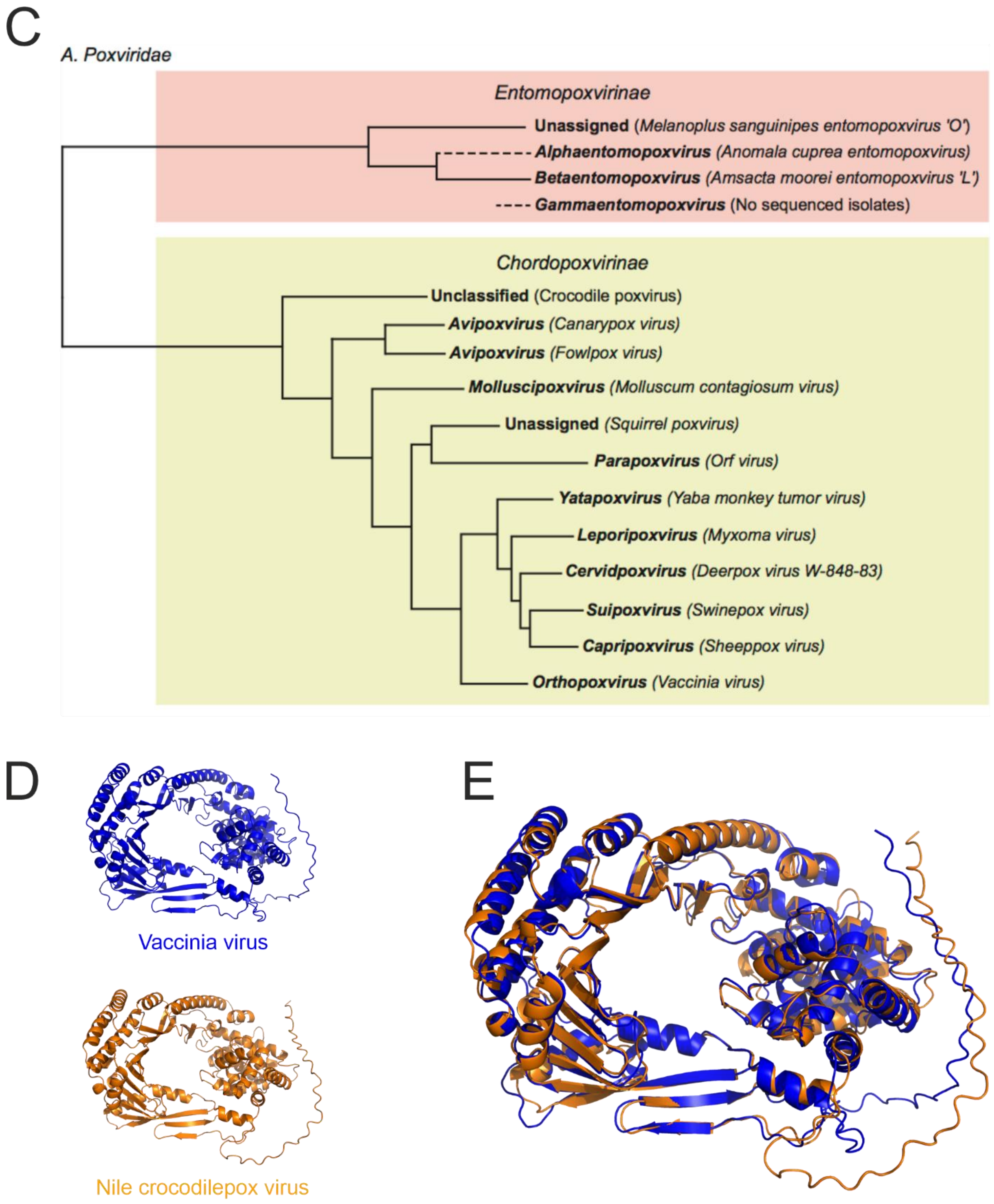

Supplementary Figure 4: Protein sequence conservation of VITF-3 among different Poxviruses. Protein sequence alignment of VITF-3l (**A**) and VITF-3s (**B**) from *Vaccinia virus* (query, UniProt Q80HV2) with Vaccinia virus (UniProt A0A6B7KEZ4), *Cowpox virus* (UniProt Q80DV2), *Taterapox virus* (UniProt Q0NP66), *Variola virus* (UniProt Q0N518), *Monkeypox virus* (UniProt Q3I8M9) and *Nile crocodile poxvirus* (UniProt Q070A5). **C** Phylogenetic tree of Poxviruses. All viral strains used for the MSA in (A) and (B) belong to the Orthopoxviruses and are closely related, except the *Nile crocodile poxvirus* (*crocodile poxvirus*). This figure was adopted from the International Committee on Taxonomy of Viruses (ICTV). Family: Poxviridae. In: Virus Taxonomy: Ninth Report of the International Committee on Taxonomy of Viruses

(2009). **D** AlphaFold predictions of VITF-3 from *Vaccinia virus* (blue) and *Nile crocodile poxvirus* (orange). **E** Alignment of the VITF-3 structures presented in (D) with similar coloring.

Supplementary Tables

Supplementary Table 1: List of DNA scaffolds used for 5'-labelling and EMSAs

Description	Sequence in 5'–3' orientation
G8R[−35–+35]_temp	CGCAGTTTATCGATTTTTATACGGATGCTCATTTTAAATTTTTGTAAATTATTTAAAGTTAAATGGCTGC
G8R[−35–+35]_bub[−5–+8]_temp	CGCAGTTTATCGATTTTTATACGGATGGAGTAAAATTTAATTTGTAAATTATTTAAAGTTAAATGGCTGC
G8R[−35–+35]_non-temp	GCAGCCATTTAACTTTAAATAATTTACAAAAATTTAAAATGAGCATCCGTATAAAAATCGATAAACTGCG
G8R[−35–+35]_bub[−5–+8]_ΔiIE_temp	CGCAGTTTATCGATTTTTATACGGATGGAGTAATTCTTAATTTGTAAATTATTTAAAGTTAAATGGCTGC
G8R[−35–+35]_ΔiIE_non-temp	GCAGCCATTTAACTTTAAATAATTTACAAAAATTCTTAATGAGCATCCGTATAAAAATCGATAAACTGCG
G8R[−35–+35]_bub[−5–+8]_ ΔiCE_temp	CGCAGTTTATCGATTTTTATACGGATGGAGTAAAATTTAATTTGTAAACATACAGAGAAACAATGGCTGC
G8R[−35–+35]_ΔiCE_non-temp	GCAGCCATTGTTTCTCTGTATGTTTACAAAAATTTAAAATGAGCATCCGTATAAAAATCGATAAACTGCG
Random[−35–+35]_bub[−5–+8]_temp	CATCATTACATAATTGTATGATAATGTTTATAATTCAGTGGCATGAAACATACAGAGAAACATCGAACTA
Random[−35–+35]_non-temp	TAGTTCGATGTTTCTCTGTATGTTTCATGCGTGACTTAATATTACATTATCATACAATTATGTAATGATG
Random[−35–+35]_bub[−5–+8]_+iIE_temp	CATCATTACATAATTGTATGATAATGTTTATAAAATAGTGGCATGAAACATACAGAGAAACATCGAACTA
Random[−35–+35]_+iIE_non-temp	TAGTTCGATGTTTCTCTGTATGTTTCATGCGTGATAAAATATTACATTATCATACAATTATGTAATGATG
Random[−35–+35]_bub[−5–+8]_+iCE_temp	CATCATTACATAATTGTATGATAATGTTTATAATTCAGTGGCATGAAATTATTTAAAGTTAATCGAACTA
Random[−35–+35]_+iCE_non-temp	TAGTTCGATTAACTTTAAATAATTTCATGCGTGACTTAATATTACATTATCATACAATTATGTAATGATG
