# Supplementary material for: Cooperative clamp-mediated promoter recognition by poxviral RNA polymerase and its TBP/TFIIB-like partner
Source: Nat Commun. 2026 Feb 18;17:1648. doi: 10.1038/s41467-026-69571-1 (PMC12917281; doi:10.1038/s41467-026-69571-1)
Supplement: Supplementary file 2 — Description of Additional Supplementary Files [file 41467_2026_69571_MOESM2_ESM.pdf]

Description of Additional Supplementary Files

Supplementary Movie 1: Supplementary Data 1

Description: Flexibility of the iPIC. Molecular morphing between the iPICd and iPICs structures.
