# Supplementary material for: Cooperative clamp-mediated promoter recognition by poxviral RNA polymerase and its TBP/TFIIB-like partner
Source: Nat Commun. 2026 Feb 18;17:1648. doi: 10.1038/s41467-026-69571-1 (PMC12917281; doi:10.1038/s41467-026-69571-1)
Supplement: Supplementary file 4 — Reporting Summary [file 41467_2026_69571_MOESM4_ESM.pdf]

nature portfolio

Corresponding author(s): Utz Fischer
Clemens Grimm

Last updated by author(s): Jan 16, 2026

Reporting Summary

Nature Portfolio wishes to improve the reproducibility of the work that we publish. This form provides structure for consistency and transparency in reporting. For further information on Nature Portfolio policies, see our Editorial Policies and the Editorial Policy Checklist.

Statistics

For all statistical analyses, confirm that the following items are present in the figure legend, table legend, main text, or Methods section.

n/a	Confirmed	
☐	☒	The exact sample size (*n*) for each experimental group/condition, given as a discrete number and unit of measurement
☐	☒	A statement on whether measurements were taken from distinct samples or whether the same sample was measured repeatedly
☒	☐	The statistical test(s) used AND whether they are one- or two-sided<br>*Only common tests should be described solely by name; describe more complex techniques in the Methods section.*
☒	☐	A description of all covariates tested
☒	☐	A description of any assumptions or corrections, such as tests of normality and adjustment for multiple comparisons
☐	☒	A full description of the statistical parameters including central tendency (e.g. means) or other basic estimates (e.g. regression coefficient) AND variation (e.g. standard deviation) or associated estimates of uncertainty (e.g. confidence intervals)
☒	☐	For null hypothesis testing, the test statistic (e.g. *F*, *t*, *r*) with confidence intervals, effect sizes, degrees of freedom and *P* value noted<br>*Give P values as exact values whenever suitable.*
☒	☐	For Bayesian analysis, information on the choice of priors and Markov chain Monte Carlo settings
☒	☐	For hierarchical and complex designs, identification of the appropriate level for tests and full reporting of outcomes
☒	☐	Estimates of effect sizes (e.g. Cohen's *d*, Pearson's *r*), indicating how they were calculated

Our web collection on statistics for biologists contains articles on many of the points above.

Software and code

Policy information about availability of computer code

Data collection	Scan software, EPSON Scan version 3.9.2.2 DE (Seiko Epson Corporation, Japan)<br>Cryo-EM, Thermo Fisher Titan Krios G3 equipped with a Falcon III camera (Thermo Fisher Scientific, USA)<br>Mass Spectrometry, Orbitrap Fusion (Thermo Fisher Scientific, USA) equipped with a PicoView Ion Source (New Objective, USA) and coupled to an EASY-nLC 1,000 (Thermo Fisher Scientific, USA)

Data analysis	Microsoft 365<br>CryoSPARC v4.2<br>PyMol version 2.5.4<br>UCSF ChimeraX version 1.3rc202111020206<br>AlphaFold 2<br>AlphaFold 3<br>COOT version 0.9.8.95<br>Phenix version 1.21<br>MaxQuant version 1.6.2.2<br>ImageJ 1.54g<br>Java 1.8.0_345 (64-bit)<br>OriginPro 2023 version 10.0.0.154<br>Jalview version 2.11.5.0<br>MEME program version 5.5.8<br>CorelDRAW 25.2.1.313

For manuscripts utilizing custom algorithms or software that are central to the research but not yet described in published literature, software must be made available to editors and reviewers. We strongly encourage code deposition in a community repository (e.g. GitHub). See the Nature Portfolio guidelines for submitting code & software for further information.

Data

Policy information about availability of data

All manuscripts must include a data availability statement. This statement should provide the following information, where applicable:

- Accession codes, unique identifiers, or web links for publicly available datasets
- A description of any restrictions on data availability
- For clinical datasets or third party data, please ensure that the statement adheres to our policy

The coordinate files generated in this study have been deposited in the Protein Data Bank under accession codes 8P0J [https://doi.org/10.2210/pdb8P0J/pdb] (iPICd), 8P0N [https://doi.org/10.2210/pdb8P0N/pdb] (iPICs), and 8P0K [https://doi.org/10.2210/pdb8P0K/pdb] (iPICCEm). The cryo-EM density data generated in this study have been deposited in the Electron Microscopy Data Bank under accession codes EMD-17334 [https://www.ebi.ac.uk/emdb/EMD-17334] (iPICd), EMD-17336 [https://www.ebi.ac.uk/emdb/EMD-17336] (iPICs), and EMD-17335 [https://www.ebi.ac.uk/emdb/EMD-17335] (iPICCEm). The structural data used in this study are available in the Protein Data Bank under accession codes 7AMV [https://doi.org/10.2210/pdb7AMV/pdb] (ePIC), 6RIE [https://doi.org/10.2210/pdb6RIE/pdb] (CCC), 3VN5 [https://doi.org/10.2210/pdb3VN5/pdb] (RNase HIII), 1YTB [https://doi.org/10.2210/pdb1YTB/pdb] (TBP/TATA-box complex), 6RFL [https://doi.org/10.2210/pdb6RFL/pdb] (complete vRNAP), 6RIC [https://doi.org/10.2210/pdb6RIC/pdb] (core vRNAP), 1AIS [https://doi.org/10.2210/pdb1AIS/pdb] (TBP/TFB core/TATA-box complex from Pyrococcus woesei), 7EGC [https://doi.org/10.2210/pdb7EGC/pdb] (RNAP II PIC), and 6EU0 [https://doi.org/10.2210/pdb6EU0/pdb] (RNAP III PIC). The mass spectrometry proteomics data have been deposited to the ProteomeXchange Consortium via the PRIDE partner repository with the dataset identifier PXD065561 [https://www.ebi.ac.uk/pride/archive/projects/PXD065561]. Any additional information required to reanalyze the data reported in this paper is available from the lead contact upon request. Source data are provided with this paper.

Research involving human participants, their data, or biological material

Policy information about studies with human participants or human data. See also policy information about sex, gender (identity/presentation), and sexual orientation and race, ethnicity and racism.

Reporting on sex and gender	n.a.
Reporting on race, ethnicity, or other socially relevant groupings	n.a.
Population characteristics	n.a.
Recruitment	n.a.
Ethics oversight	n.a.

Note that full information on the approval of the study protocol must also be provided in the manuscript.

Field-specific reporting

Please select the one below that is the best fit for your research. If you are not sure, read the appropriate sections before making your selection.

☒ Life sciences ☐ Behavioural & social sciences ☐ Ecological, evolutionary & environmental sciences

For a reference copy of the document with all sections, see nature.com/documents/nr-reporting-summary-flat.pdf

Life sciences study design

All studies must disclose on these points even when the disclosure is negative.

Sample size	A standard sample size of three biological replicates was used for in vitro experiments.
Data exclusions	No data was excluded from the analysis.
Replication	For functional experiments, the data of three biological replicates are provided in source data.
Randomization	n.a.
Blinding	n.a.

Reporting for specific materials, systems and methods

We require information from authors about some types of materials, experimental systems and methods used in many studies. Here, indicate whether each material, system or method listed is relevant to your study. If you are not sure if a list item applies to your research, read the appropriate section before selecting a response.

Materials & experimental systems

n/a	Involved in the study	
☐	☒	Antibodies
☐	☒	Eukaryotic cell lines
☒	☐	Palaeontology and archaeology
☒	☐	Animals and other organisms
☒	☐	Clinical data
☒	☐	Dual use research of concern
☒	☐	Plants

Methods

n/a	Involved in the study	
☒	☐	ChIP-seq
☒	☐	Flow cytometry
☒	☐	MRI-based neuroimaging

Antibodies

Antibodies used	H-20 antibody, A kind gift from Reinhard Lührmann<br>Anti-HA.11 Epitope Tag Antibody, BioLegends, Cat# 901516 (Previously Covance catalog# MMS-101R), Clone 16B12<br>Anti-VITF-3 (polyclonal), immunoGlobe Antikörpertechnik GmbH
Validation	H-20 antibody, Bochnig et al., 1987<br>Anti-HA.11 Epitope Tag Antibody, Validated applications according to the vendor: western blot (WB), immunocytochemistry (ICC), immunoprecipitation (IP), and flow cytometry (FC)

Eukaryotic cell lines

Policy information about cell lines and Sex and Gender in Research

Cell line source(s)	HeLa S3, Sigma-Aldrich, Cat#87110901, female<br>African green monkey kidney fibroblasts (CV-1), ATCC, Cat#CCL-70, male
Authentication	All cell lines are commercially available.
Mycoplasma contamination	All cell lines were tested negative for mycoplasma contamination.
Commonly misidentified lines (See ICLAC register)	n.a.

Plants

Seed stocks	n.a.
Novel plant genotypes	n.a.
Authentication	n.a.
